# Supplementary material for: Fibroblast growth factors (FGFs) prime the limb specific Shh enhancer for chromatin changes that balance histone acetylation mediated by E26 transformation-specific (ETS) factors
Source: eLife. 2017 Sep 26;6:e28590. doi: 10.7554/eLife.28590 (PMC5659820; doi:10.7554/eLife.28590)
Supplement: Supplementary file 1. — (B) List of oligos used for 3C and 4C analysis. [file elife-28590-supp1.docx]

**Supplementary information**

**ST1 (Supplementary File 1A)**

| **Description** | **5'-3' Sequence** |
| --- | --- |
| Mouse SHH qRT-PCR | TCCACTGTTCTGTGAAAGCAG |
|  | GGGACGTAAGTCCTTCACCA |
| Mouse GABPα qRT-PCR | CGGGGAGAAATTCTTTGGA |
|  | CTTGGCTGGCCCCAAAACATA |
| Mouse ETV4 qRT-PCR | CAGCAGGAAGCCACCACT |
|  | GGACTTGATGGCGATTTGTC |
| Mouse HDAC2 qRT-PCR | CTCCACGGGTGGTTCAGT |
|  | CCCAATTGACAGCCATATCA |
| Mouse HDAC1 qRT-PCR | TGGTCTCTACCGAAAAATGGAG |
|  | TCATCACTGTGGTACTTGGTCA |
| Mouse GAPDH qRT-PCR | GGGTTCCTATAAATACGGACTGC |
|  | CCATTTTGTCTACGGGACGA |
| Mouse SHH RT-PCR | CGGCAGATATGAAGGGAAGAT |
|  | GGATTCATAGTAGACCCAGTC |
| Mouse dHAND RT-PCR | CGACACCAAACTCTCCAAGATC |
|  | AACCGGATGATCCAACTGCAAG |
| Mouse GREMLIN RT-PCR | GCACCAATCTCGTTCAGATAC |
|  | AACCGGATGATCCAACTGCAAG |
| Mouse ALX4 RT-PCR | CTTCACTAGCTACCAGCTGGAA |
|  | ATTGTTGCCAATCCAGGATGG |
| Mouse HGRPT RT-PCR | CAGTCAACGGGGGACATAAAAG |
|  | TCAACTTGCGCTCATCTTAGGC |
| ZRS 3’LR ChIP | AGAAGAGAGTAGGAAGTCAGCCT |
|  | GAGCCTTTCTCTTGCCTGTATTT |
| ZRS 5’ST ChIP | GACAGCAACATCCTGACCAA |
|  | CATGTGTGAGGTTCTGGACAC |
| SHH promoter ChIP | GAGACCCAACTCCGATGTGT |
|  | GCCTGGCTCTTTCTCTTCCT |
| Control region ChIP | TCTGCTCATCCAGTGAGGTG |
|  | TGACCTCCACAAGAGCACTG |
| Rbm33 intron 1 | GGGTTGTCAGAAGGAACCATT |
|  | GGATTCTCAGAGCTTTTGCAC |
| Mnx1 intron 1 | ACATGCAGCGTCTGTGTAGG |
|  | CAGCTTGCCCCTATCCAAG |
| DUSP6 qRT-PCR | CCTGAGGCCATTTCTTTCATAGA |
|  | GCAGCTGGCCCATGAAGTTGAAGT |

List of oligos used for qRT-PCR, q-PCR and ChIP.

**ST2 (Supplementary File 1B)**

| 3C site -1 | GCCAAGCAGTTAGAAGGTTCA |
| --- | --- |
| 3C site 1 | TCTTCATTCCAGCCTGACCT |
| 3C site 2 | TGGAGAGCTTGTGAGACAGG |
| 3C site 5 | CTCACTCATTGCCAGTTATCTCACCC |
| 3C ZRS bait | AAACACACACCGCCCAATG |
| ZRS probe | TTGCAGCCATTCACTTGCACTGTCA |
| Site -1 digestion efficiency | TGCCCTGGTGCTTTAGTGTA |
| Site 1 digestion efficiency | CCTATCCATCCTACCCTCGC |
| Site 2 digestion efficiency | GGGTTAACATCAGAAGACAAGCT |
| ZRS digestion efficiency | AGCTTGCTTTTGTTGTAGGGA |
| 4C *Hin*dIII primer | GGGGAACTGATCACAAGA |
| 4C MlucI primer | CATCTTTTTCTTGCAGGTGT |

List of oligos used for 3C and 4C analysis.
